# Supplementary material for: Effectiveness of modifications to preadjusted appliance prescriptions based on racial dental characteristics assessed by the ABO Cast-Radiograph Evaluation: A propensity score matching study
Source: PeerJ. 2021 Jan 15;9:e10605. doi: 10.7717/peerj.10605 (PMC7812923; doi:10.7717/peerj.10605)
Supplement: Supplemental Information 1 [file peerj-09-10605-s001.docx]

TableV.Independent samples t-test for the buccolingual inclination of anterior teeth and the treatment duration

| Type | Mean | | Stand Deviation | | Mean Difference | 95% Confidence Interval | | P value |
| --- | --- | --- | --- | --- | --- | --- | --- | --- |
|  | Chinese | Roth | Chinese | Roth |  | Lower | Upper |  |
| U1/SN | 105.1 | 106.0 | 7.4 | 6.9 | -0.8 | -4.5 | 2.9 | 0.68 |
| L1/MP | 96.0 | 95.0 | 6.4 | 6.0 | 1.0 | -2.2 | 4.3 | 0.52 |
| Treatment Duration | 22.4 | 23.5 | 3.6 | 3.0 | -1.1 | -2.8 | 0.6 | 0.21 |

Independent samples t-test, * *p*<0.05, statistical significance was set at *p*<0.05
